# The two chytrid pathogens of amphibians in Eurasia—climatic niches and future expansion

**DOI:** 10.1186/s12862-023-02132-y

**Published:** 2023-06-27

**Authors:** Dan Sun, Gajaba Ellepola, Jayampathi Herath, Madhava Meegaskumbura

**Affiliations:** 1 Guangxi Key Laboratory for Forest Ecology and Conservation, College of Forestry, Guangxi University, Nanning, Guangxi, 530000 People’s Republic of China; 2 Department of Zoology, Faculty of Science, University of Peradeniya, Peradeniya, Kandy, 20400 Sri Lanka

**Keywords:** Amphibian decline, *Batrachochytrium*, Climate change, Disease spread, Pathogens, Realized climatic niche

## Abstract

**Background:**

Climate affects the thermal adaptation and distribution of hosts, and drives the spread of Chytridiomycosis—a keratin-associated infectious disease of amphibians caused by the sister pathogens *Batrachochytrium dendrobatidi* (*Bd*) and *B. salamandrivorans* (*Bsal*). We focus on their climate-pathogen relationships in Eurasia, the only region where their geographical distributions overlap. Eurasia harbours invaded and native areas of both pathogens and the natural habitats where they co-exist, making it an ideal region to examine their environmental niche correlations. Our understanding of how climate change will affect their distribution is broadened by the differences in climate correlates and niche characteristics between *Bd* and *Bsal* in Asia and Europe. This knowledge has potential conservation implications, informing future spread of the disease in different regions.

**Results:**

We quantified the environmental niche overlap between *Bd* and *Bsal* in Eurasia using niche analyses. Results revealed partial overlap in the niche with a unique 4% of non-overlapping values for *Bsal*, suggesting segregation along certain climate axes. *Bd* tolerates higher temperature fluctuations, while *Bsal* requires more stable, lower temperature and wetter conditions. Projections of their Realized Climatic Niches (RCNs) to future conditions show a larger expansion of suitable ranges (SRs) for *Bd* compared to *Bsal* in both Asia and Europe, with their centroids shifting in different directions. Notably, both pathogens' highly suitable areas in Asia are expected to shrink significantly, especially under the extreme climate scenarios. In Europe, they are expected to expand significantly.

**Conclusions:**

Climate change will impact or increase disease risk to amphibian hosts, particularly in Europe. Given the shared niche space of the two pathogens across available climate gradients, as has already been witnessed in Eurasia with an increased range expansion and niche overlap due to climate change, we expect that regions where *Bsal* is currently absent but salamanders are present, and where *Bd* is already prevalent, may be conducive for the spread of *Bsal.*

**Supplementary Information:**

The online version contains supplementary material available at 10.1186/s12862-023-02132-y.

## Background

Global amphibian populations are continuing to decline, leading to species extinction, and the situation is predicted to worsen in the future [1–3]. These declines are the result of several factors, such as habitat change [4], pollutants and pesticides [2, 5], climate change [6, 7], and infectious diseases [8–10]. Climate has an effect on wildlife, both directly (e.g., thermal adaptation and species distribution), and indirectly; it is a key driver of disease spread, as it regulates temperature and humidity regimes [11, 12]. It is also indicated that the climate change may alter dynamics of species susceptibility by breaking the balance or long-term coexistence between hosts and pathogens, triggering disease shifts [13–15]. Recent studies have predicted that climate change will likely increase the prevalence of infectious pathogens, thus posing a risk of disease while affecting new hosts [16]. Therefore, it is essential to understand the ecological niche correlates of amphibian pathogens and their dispersal patterns under climate variations, to facilitate amphibian conservation.

Chytridiomycosis has caused the greatest amphibian population decline and species extinction in the world, posing an ongoing threat to biodiversity [8, 17]. The disease is caused by two sister chytrid pathogens, *Batrachochytrium dendrobatidi* (*Bd*) [18, 19] and *B. salamandrivorans* (*Bsal*) [20], which has been thought to originated in East Asia during the late Cretaceous or early Paleogene [17, 21]. Asian hosts are considered resistant to Chytridiomycosis due to their long interactions with the pathogens [22, 23], yet they have spread to other regions such as Europe, the Americas and Australia [21, 24], affecting over 500 species of anurans and urodelans [21, 25].

*Bd* has a broader worldwide distribution than *Bsal*, which has not been found in the Americas but is relatively restricted in Eurasia (Asia and Europe) [26, 27]. Notably, both pathogens co-occur in natural habitats of East and Southeast Asia and Western Europe [26, 28, 29]. In Asia, seventeen countries have reported the presence of *Bd* [30–32], with four also reporting *Bsal* [17, 28, 33]. In Europe, *Bd* has been detected in about 27 countries (amphibian disease web portal: https://amphibiandisease.org), and *Bsa*l in four (http://bsaleurope.com/), with a more limited distribution.

In Eurasia, the pathogens have a considerable degree of spatial overlap, but there are also regions where they do not overlap [28, 29]. Complex biotic and abiotic factors and the differences in ecological requirements of the pathogens are the likely cause for these non-overlapping regions. Abiotic factors, in particular thermal parameters, likely play a role; laboratory studies suggest *Bd* prefers slightly higher temperatures (17–25℃) compared to much cooler conditions for *Bsal* (10–15℃) [20, 34]. *Bd* is a host-generalist that infects all orders of amphibians [30], while *Bsal* is a host-specialist, mainly infecting Urodela (salamanders and newts). However, the realized climatic niches (RCNs) of the two pathogens have not been comprehensively compared. Understanding the differences between their climate niches can help predict how they will respond to climate change, allowing mitigation actions to be taken to control their spread.

Climate warming is presumably shaping the potential suitable habitats of the two pathogens, *Bd* and *Bsal*. Studies for *Bd* based on previous versions of climate models suggest climate change will likely enhance expansion dynamics in temperate and cooler regions [16, 35], but *Bsal*'s future range expansion is still poorly understood. Updated versions of the Coupled Model Inter-comparison Project Phase 6 (CMIP6) climate models putatively allow more accurate predictions of species distribution when compared to the previous version [36–38]. Given that the region of our interest includes both native and invaded areas, Eurasia is an ideal region to compare the shared environmental niche correlations between *Bd* and *Bsal* and evaluate how the two pathogens respond to climate change on a larger scale. In addition, the dissimilar climatic conditions in Asia and Europe, as well as the climatic niche requirements of *Bd* and *Bsal*, coupled with various climate scenarios, may lead to divergent dispersal patterns in the future. These variations could have critical implications for amphibian conservation, especially in areas with a high diversity of urodelans, where *Bsal* has not yet established itself.

We aimed to compare the ecological niches of *Bd* and *Bsal* in Eurasia and anticipate their range shifts in response to climate change. Patterns to formulate a hypothesis included: 1) differences of host features of both pathogens [8, 39], 2) differential environmental conditions in native and invaded areas in Eurasia [17, 21] and, 3) distributional overlap of pathogens in Asia and Europe [17, 28, 40]. Based on the observed patterns, we hypothesized that the two pathogens have subtle differences in their RCNs to occupy respective habitats, with *Bd* having a broader niche range. The hypothesis if true would predict that the two pathogens would respond differently to future climate changes and range shifts between them would differ under various climatic scenarios.

## Results

### Realized niche overlaps between *Bd* and *Bsal*

**Fig. 1:**
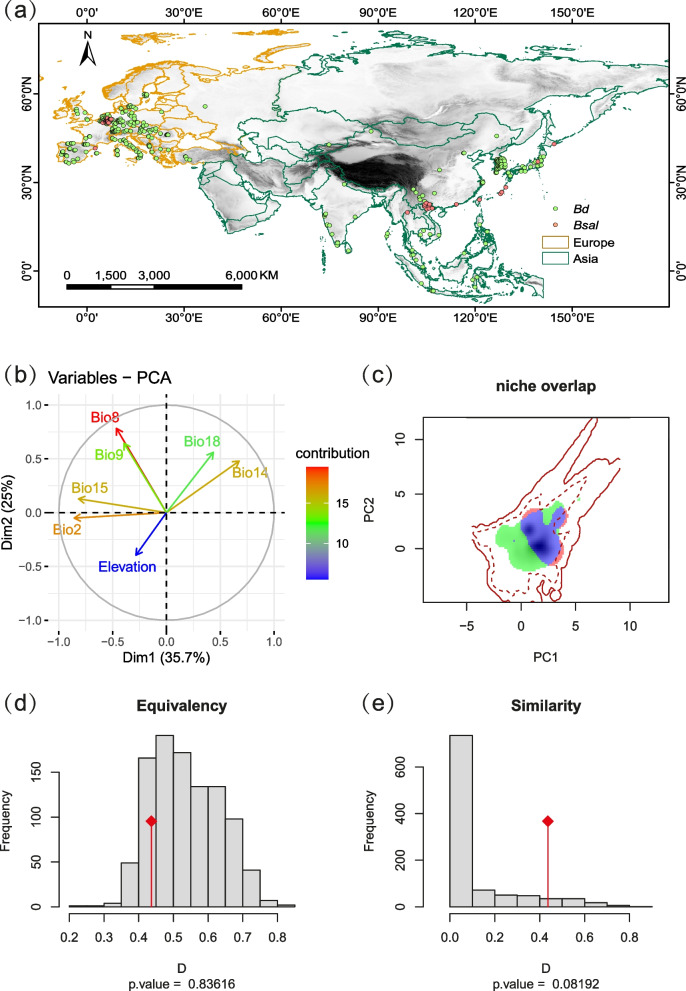
Distribution of *Bd* and *Bsal* and niche overlap analysis. Localities positive for *Bd* (green dots) and *Bsal* (red dots) on a background of altitudinal gradients are shown in **a**. The contributions of environmental variables to the first two PC axes are presented in **b** (standard definitions for each variable provided in Additional file 1: Table S1). A visualization of occupied and available niche space for the two pathogens is provided in **c**, based on the first two axes of a principal component analysis. Solid and dashed contour lines represent 100% and 50% of the available environment in Eurasia, respectively. The green, red and blue filled areas indicate the occupied niches of *Bd*, *Bsal* and overlap, respectively, with deeper colors representing higher overlap. Histograms **d**-**e** display the expected distribution of Schoener's *D* index values for the niche equivalence and niche similarity tests, with red diamonds indicating the actual observed niche overlap

The multivariate principal component analysis comparing the niches of *Bd* and *Bsal* in Eurasia (Fig. 1) revealed that the two pathogens exhibited environmental niche correlations. They occupied different niche spaces, and segregated based on factors such as mean diurnal range (Bio2), precipitation of the driest month (Bio14), precipitation seasonality (Bio15), precipitation of warmest quarter (Bio18) and elevation.

**Fig. 2:**
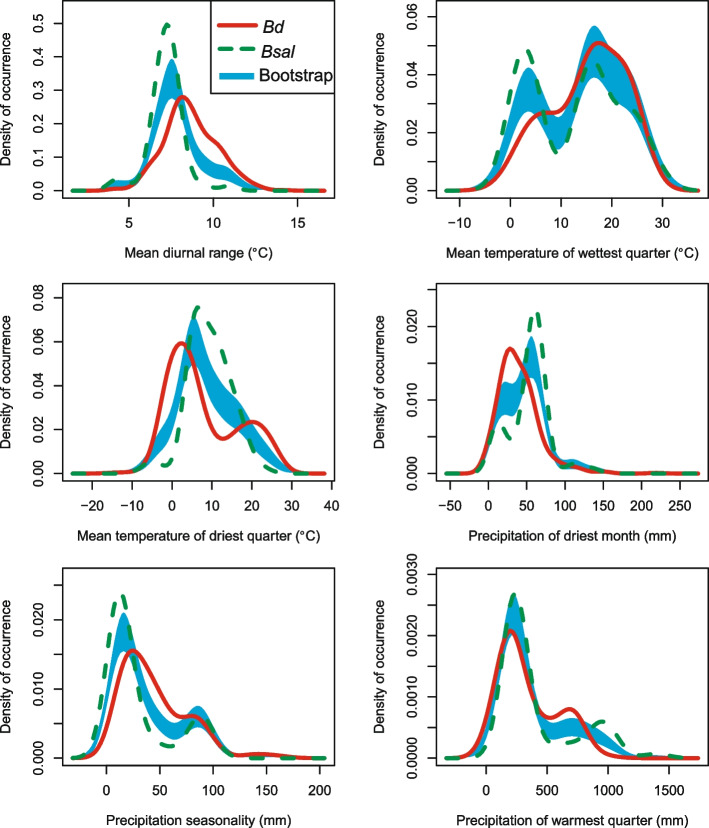
Density distributions of *Bd* and *Bsal* occurrences along univariate axes. The hypothesis of niche equivalency is rejected when density distributions (*Bd*: red solid line and *Bsal*: green dotted line) exceed the estimated confidence limits (blue bands)

However, the highest proportion of niche overlap for *Bd* and *Bsal* occurred along PC1 (Fig. 1c) with moderate values of *D* = 0.4365 and *I* = 0.6269. The niche equivalency and similarity between the pathogens were rejected due to subtle differences in the niches (Fig. 1d-e, Fig. 2). The niche stability value (environmental niche occupied by both *Bsal* and *Bd*) was high (0.9559) indicating that a large proportion of the niche space overlapping. Out of the total niche space, *Bd* occupied 0.2657 that did not overlap with *Bsal*, whereas *Bsal* occupied a distinct niche space of 0.0441.

Univariate comparison of RCNs demonstrated varying degrees of niche overlap and differences between the two pathogens across all variables (Fig. 2). Notably, *Bd* had a broader diurnal range (Bio2) and mean temperature in driest quarter (Bio9) than *Bsal*. Distribution trends in temperature parameters suggested *Bd* could tolerate higher temperature fluctuations while *Bsal* had a relatively stable temperature regime. The comparison along the precipitation of the driest month variable highlighted *Bsal*'s preference for slightly wetter conditions than *Bd*.

### Performance of MaxEnt models and variable contributions

The MaxEnt models of the two pathogens recorded high AUC and TSS scores; *Bd*'s AUC was 0.948 ± 0.002 and TSS was 0.795 ± 0.019, and *Bsal*'s AUC and TSS were 0.984 ± 0.006 and 0.899 ± 0.035 respectively. This demonstrates the models' robustness and adequacy for predicting the climate suitability for *Bd* and *Bsal*.

Analysis of MaxEnt (Table 1; Additional file 2: Fig. S1) indicated variations in major bioclimatic variables between the two pathogens. Temperature-related parameters were the major contributors in the *Bd* model, with similar contributions from precipitation-related variables in the *Bsal* model.

**Table 1:** Contributions of bioclimatic variables to the MaxEnt models

Pathogen	Percentage (%)	Bio2	Bio8	Bio9	Bio14	Bio15	Bio18
*Bd*	Contribution	15.5	9.9	41.8	24.8	3.4	4.7
	Permutation	11.1	11.8	50.1	10.3	7.4	9.4
*Bsal*	Contribution	27.8	0.2	20.6	6.6	33.9	10.9
	Permutation	18.2	2.3	29.7	1.4	22.8	25.6

### Centroid distribution shifts of the two pathogens

**Fig. 3:**
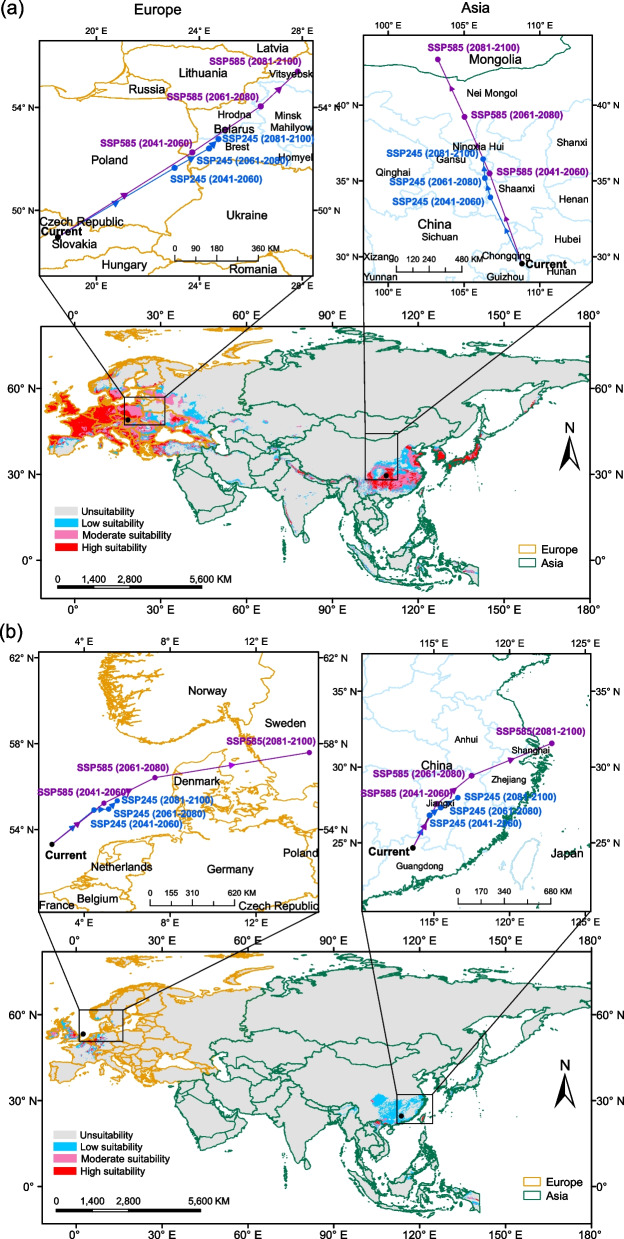
Current climatic suitability and centroid shifts of *Bd* and *Bsal*. The current potential distribution (map) and shifts in centroid distribution (the boxes above maps) in Europe and Asia under future time periods (2041–2060, 2061–2080, 2081–2100) based on SSP245 and SSP585 climate scenarios for *Bd* **a** *Bsal* **b**

The predicted future distribution changes reflected the differences in climatic niches of the two pathogens. The shifting of centroids in suitable ranges (SRs) was larger for *Bd* than *Bsal*, and larger centroid shifts occurred under SSP585 climate scenario rather than SSP245 (Fig. 3). These patterns held for both Asia and Europe. Additionally, the centroids of both pathogens in Europe shifted towards the east of their current ranges; the centroid of *Bd* in Asia moved towards northern latitudes (Fig. 3a), while that of *Bsal* was expected to move towards East Asia, suggesting they may behave differently in Asia in response to climate change (Fig. 3b).

### Changes in suitable habitats

**Fig. 4:**
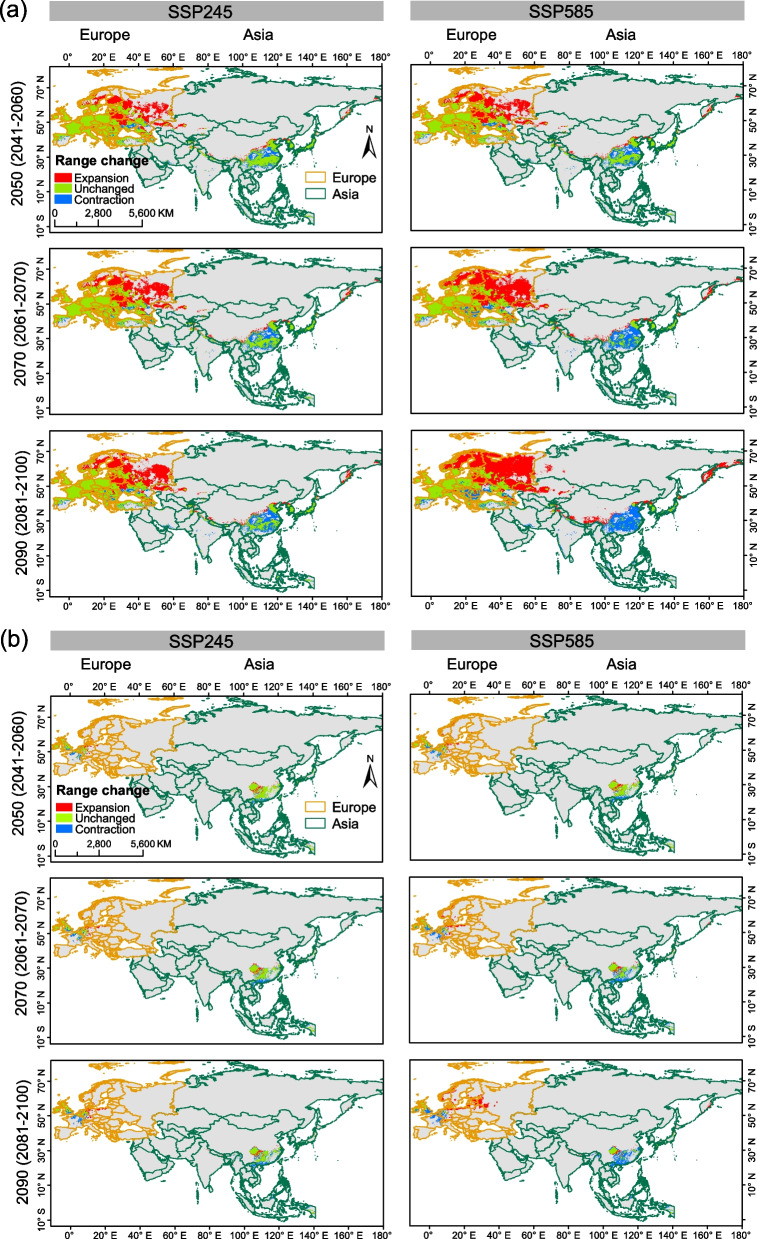
Future range changes of *Bd* **a** and *Bsal* **b** in Europe and Asia. The changes in distributions are depicted for three future periods (2041–2060, 2061–2080, and 2081–2100) with two climate scenarios (SSP245 and SSP585)

Our analyses suggest that the overall geographic range size of *Bd* and *Bsal* would gradually increase in Eurasia during 2041–2060, 2061–2080 and 2081–2100 periods (Additional file 1: Table S2). In Europe, there was a marked expansion in suitable areas for both pathogens compared to their range size in Asia. In Asia, there was a substantial contraction in their range size in the southern regions across lower latitudes (Fig. 4).

In Europe, the current distributions of both pathogens are predicted to be concentrated in temperate and cold climate regions with weak dry seasons and warm summers (Fig. 3; Additional file 2: Fig. S2). The ensemble model identified more areas in Eastern Europe as potential suitable habitats for both pathogens compared to the single MaxEnt model (Additional file 2: Fig. S3). A distinct expansion in 2081–2100 period under SSP585 climate scenario was shown for both pathogens (Fig. 4, Additional file 2: Fig. S4). However, compared to *Bsal*, *Bd* is expected to have greater range shifts, and most regions would likely hold optimal climatic conditions for a long period (Fig. 4).

In Asia, current distributions for both pathogens were across eastern mountainous coastal areas and high altitudes of temperate climate regions (Fig. 3; Additional file 2: Fig. S2). The ensemble models predicted more suitable habitats in Southeast Asia (Additional file 2: Fig. S3). Future predictions suggest that their ranges would expand into the Hengduan Mountains, North China Plain, and coastal mountainous regions in China. *Bsal*'s future distribution was fragmented and concentrated in higher altitudes, with Southern and Central China becoming more suitable than *Bd* (Fig. 4).

**Fig. 5:**
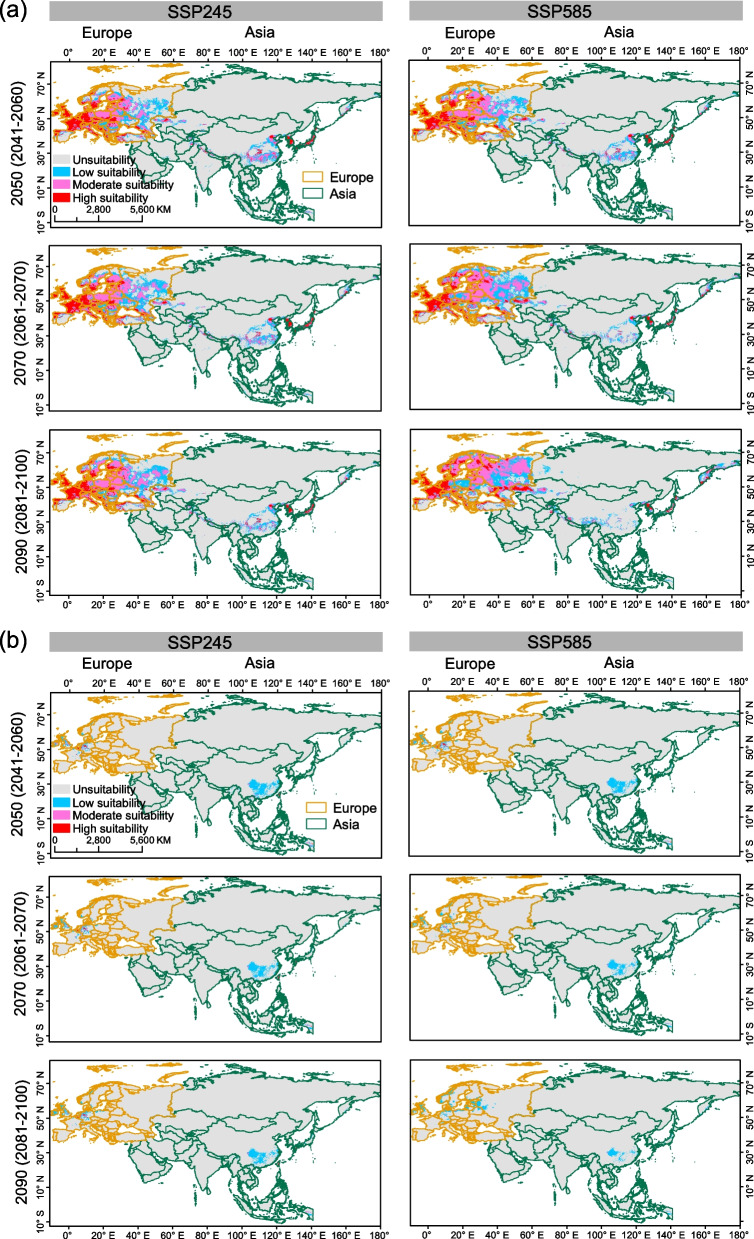
Future climatic suitability of *Bd* **a** and *Bsal* **b**. Different colors represent classes of future habitat suitability; low suitability, moderate suitability and high suitability. Suitability categories are looked under two climate scenarios (SSP245 and SSP585) across future three periods (2041–2060, 2061–2080, 2081–2100)

A major proportion of range expansion for both pathogens (Fig. 4) was reflected more in areas currently classified as low and/or moderately suitable. We therefore examined changes in ‘highly suitable areas’ in Asia and Europe (Fig. 5). Our results showed that the highly suitable areas for *Bd* in Asia would shrink (Additional file 2: Fig. S5) despite shifting towards East Asia (Fig. 5a). *Bsal*'s extension of highly suitable habitats would become less suitable (Fig. 5b) despite the overall expansion shown in Fig. 4. In Europe, *Bd* was projected to have low and moderate suitability regions in Eastern Europe, while Western and Central Europe were projected to maintain high suitability (Fig. 5a). *Bsal* was expected to have a wider extension in low suitability habitats in the future, while highly and moderately suitable areas were projected to decrease (Fig. 5b, Additional file 2: Fig. S5).

### Future niche variations between *Bd* and *Bsal*

The niche breadth (*B* value) of both pathogens is likely to expand under future climate scenarios (Table 2). Specifically, *Bd*'s niche breadth is expected to be larger than that of *Bsal*. The niche overlap between the two pathogens was projected to increase during the three future periods considered. Range overlap was also predicted to gradually increase under SSP245 climate scenario.

**Table 2:** Estimated niche breadth (*B*), niche overlap (*D* and *I*) and range overlap. The niche values of *Bd* and *Bsal* were calculated based on the maps of predicted habitat suitability under SSP245 and SSP585 climate scenarios. Range overlap was estimated based on the threshold value (0.6) of high suitability

				SSP245			SSP585	
		Current	2041–2060	2061–2080	2081–2100	2041–2060	2061–2080	2081–2100
*B*	*Bd*	0.2031	0.2522	0.2636	0.2707	0.2623	0.2898	0.3267
	*Bsal*	0.0768	0.1027	0.1128	0.1189	0.1109	0.1375	0.1690
*D*		0.4003	0.4306	0.4493	0.4583	0.4438	0.4843	0.5118
*I*		0.7054	0.7340	0.7486	0.7561	0.7442	0.7701	0.7881
Range overlap		0.6722	0.7002	0.7135	0.7227	0.6831	0.4646	0.2773

## Discussion

Eurasia is an important region for distribution of *Bd* and *Bsal* [17, 21], as they co-exist there under suitable climatic conditions [28, 29]. We studied their RCNs and used future climate models to assess their habitat suitability and niche variations. We also considered how range shifts might differ between Asia and Europe given the different climatic factors in each region.

### Niche overlap and climate preferences between *Bd* and *Bsal*

Our results suggest that *Bd* and *Bsal* share a similar environmental niche space. Their overlapping climatic space is associated with mean temperatures of wettest and driest quarters (Fig. 1b and c). *Bsal* showed highest occurrence densities than *Bd* in all variables except for mean temperature of wettest quarter (Fig. 2), indicating that its optimum conditions for *Bsal* may be suitable for *Bd* as well. Notably, *Bsal* is able to occupy under highly humid conditions as reflected by higher occurrence densities under precipitation of driest month and precipitation of warmest quarter (Fig. 2). Therefore, *Bsal*'s environmental associations and high virulence [20, 41] suggest it may spread to *Bd*-present habitats with salamanders in the future. Overall, wet conditions appear to shape *Bsal*'s occurrence, as shown by other studies [39, 42].

### Current suitable habitats and future range shifts under scenarios of climate change

MaxEnt-based models agree with most prior studies, but differ slightly in *Bd* and *Bsal*'s distributions. *Bd* is predicted to spread across Europe, while past models suggested less suitability in Northern-Central-Eastern Europe [43–46], the increased number of occurrence records added in the current analysis may have resulted in this difference. *Bsal*'s model predicts a higher distribution in Western-Central Europe, while region-scale studies predicted a more restricted area in southern Germany [40, 47, 48]. For Asia, both *Bd* and *Bsal* have potential distributions concentrated in Southeast-East Asia (Fig. 3) but previous studies suggested Central Asia, South Korea [35] and Kamchatka [43–45] as different SRs for *Bd.* Under future climate scenarios, range contractions of *Bd* are predicted in southern China and Southeast Asia due to warmer temperatures and excessive rainfall [49]. Previous estimates suggested *Bd* would decrease and be restricted in Europe, but our study found a broader distribution in Europe. These discrepancies in future predictions could be attributed to the use of varying spatial ranges used in different models [30, 50]. Variations in species distribution models are expected due to several factors, such as differences in spatial resolutions, predictor variables used in the analyses, and the number of occurrence records utilized. Furthermore, some studies utilize more predictors, such as amphibian species richness, which can also contribute to differences in predictions [35, 45].

*Bd* is likely to track climate change better than *Bsal*, due to its generalist nature [51], which could explain the differences in range expansion between the two pathogens (Figs. 4 and 5). Many of *Bsal*'s habitats will become less suitable due to climate change, but habitats for *Bsal* will remain highly suitable in Germany and Belgium. The centroid distribution shifts of *Bd* and *Bsal* in Asia (Fig. 3) suggest that *Bd* will move northward towards optimal temperature conditions and *Bsal* towards wetter and cooler conditions in East Asia, as will their hosts.

### Implications in amphibian conservation

Climate change could impact the range expansion and niche breadth of *Bd* and *Bsal*, potentially threatening vulnerable amphibians across Eurasia (Fig. 4 and Table 2). In Asia, amphibians may shift towards northern regions [52, 53], and *Bd* may also expand its range. As global warming continues, amphibian hosts adapting to cooler climates may be at increased infection risk [54] as temperature can influence both the kinetics of host–pathogen interactions and act as a host stressor [11]. In case where certain areas with suitable climates remain unaffected by chytrid pathogens, such as Switzerland in Europe and Himalaya highlands in Asia, should be monitored on a long-term basis. If they continue to remain free of pathogen infiltration over time [55], it would call for further studies to understand why such patterns are being observed.

## Conclusions

Our analysis across Eurasia revealed shared environmental conditions between the two chytrid pathogens and moderate niche overlap, with *Bd* having a broader RCN than *Bsal*. Our study identifies areas of potential high-priority for monitoring the spread of the chytrid fungi.

## Methods

### Study area

We delimited a large area covering Eurasia (*Xmin* = 14.28S and *Xmax* = 180.00; *Ymin* = 13.00W and *Ymin* = 90.00E, WGS84) to capture all *Bd* and *Bsal* records in the region. This includes Asia (42,968,070.93 km^2^) and Europe (10,631,747.53 km^2^). We downloaded pathogen range shapefiles from http://tapiquen-sig.jimdofree.com for use in subsequent analyses.

### Occurrence records

To model the distribution of *Bd* and *Bsal*, we obtained occurrence records of their amphibian hosts in the wild (excluding those in captivity). *Bd* coordinates were from the *Bd*-Maps Legacy Project (https://amphibiandisease.org/projects/, accessed Jun 15 2021) and *Bsal* records from relevant literature [17, 24, 28, 33, 40, 56, 57]. We applied the nearest neighbor joining index to reduce the effect of spatial autocorrelation due to sampling bias [58]. The final positive records consisted of 446 *Bd* and 57 *Bsal* observations (Fig. 1; Additional file 1: Table S3).

### Environmental variables

We initially downloaded 19 bioclimatic variables and the elevation variable from WorldClim (http://www.worldclim.org/) at a spatial resolution of 2.5 arc minutes (~ 5 × 5 km2) for the period 1970–2000 [59]. To account for multicollinearity [60], we calculated correlations between variables in ENMTools [61] and visualized them in a Pearson correlation matrix (Additional file 2: Fig. S6). We then selected six non-correlated bioclimatic variables with Pearson’s r >|0.7|, plus elevation, for further analysis [62] (Additional file 1: Table S1). These variables were used to characterize climate niches, analyze niche overlap and predict current distribution.

To reduce uncertainty in predictions of future distribution patterns, we downloaded six bioclimatic layers representative of three future time periods (2041–2060, 2061–2080, and 2081–2020). We used the latest CMIP6 models with shared socioeconomic pathways (SSPs) to enhance confidence of the outcomes [36]. Different Global Climate Models (GCMs) can affect the predicted distributions of species differently [63, 64], so we used multiple GCMs (BCC-CSM2-MR, CNRM-CW6-1, CNRM-ESM2-1, CanESM5, IPSL-CM6A-LR, MIROC-ES2L, MIROC and MRI-ESM2-0) for our models. We employed two representative concentration pathways (SSP245 and SSP585, resulting in 2100 radiative forcing levels of 4.5 W/m^2^ and 8.5 W/m^2^, respectively) [37] to represent moderate and pessimistic climate change scenarios [38].

### Niche overlap analyses

We implemented a quantitative approach to evaluate environmental niche correlates between *Bd* and *Bsal* based on ecological factors associated with pathogen presence. We performed the realized niche analysis of the two pathogens using “ecospat” package [65], where the first two axes of ordination framework were gridded into 100 × 100 cells depicting the climatic space. This permitted us to test whether differences of observed niches result from partial niche filling or niche expansion, as environmental availability is accounted for during ordination. To quantitatively compare the niches of the two pathogens, we used the principal component analysis to extract environmental variables (Additional file 1: Table S1) of each pathogen and used a smooth kernel density function to determine the ‘smoothed’ density of occurrences in each cell in the niche space [66]. We used Schoener's *D* metric and Hellinger's-based *I* index to quantify niche overlap and 1000 repetitions for generating a null distribution to analyze niche equivalence and similarity [66–68]. The scores for *D* and *I* index range from 0 (no overlap) to 1 (complete overlap). Niche stability, expansion, and unfilling between *Bd* and *Bsal* were calculated based on each cell in the ordination using “ecospat” package [65]. We performed a bootstrap hypothesis test of equality, based on a single environmental variable occupied by each pathogen using “sm” package [69], to compare their realized niches in a univariate space.

### Distribution modeling

We used MaxEnt-based species distribution models (SDMs) with a presence-only method, ensemble techniques, and an analysis of centroid shift and climatic suitability change for each pathogen in both Europe and Asia.

We predicted and quantitatively calculated the extent and changes of habitat suitability for the two pathogens, *Bd* and *Bsal*, under current climate and two future climatic scenarios (SSP245 and SSP585). The maximum entropy algorithm (MaxEnt) was selected to create habitat suitability models based on their occurrence records in Eurasia, as it is particularly suited for presence-background algorithms by comparing the available environmental conditions in the background with the conditions favorable to species occurrences [70–72].

To reduce model over-fitting and model complexity in trained current and future models [60], we performed relevant analysis using the “kuenm” package [73] and generated all possible combinations of regularization multipliers (RM: ranging from 0.0 to 4.0 in 0.1 steps) with all combinations by including the linear (L), quadratic (Q), product (P), threshold (T), and hinge (H) features. Among all created models, the model with the lowest AICc score was selected as the optimal model. Therefore, the model with the combination of QPTH feature and regularization multiplier of 1.2 was used to predict *Bd* distribution and the model with the combination of T feature and regularization multiplier of 1.0 was used to predict *Bsal* distribution. We conducted a MaxEnt analysis by using the maximum training sensitivity plus the specificity threshold (MTSS), with a training set consisting of 25% occurrence coordinates, ten replicates, and cloglog outputs [74, 75]. The Area Under the receiver operating characteristic Curve (AUC) and true skill statistic (TSS) were used to evaluate model performance [70, 76]. The model performance was evaluated as ‘good’ when the value of AUC was above 0.9 [77] and the value range of TSS between 0.6 and 0.8 was regarded as useful while it was considered good or excellent when value was over 0.8 [76].

We converted the above results from MaxEnt models into binary habitat suitability (suitable habitats and unsuitable habitats) to reclassify it into four categories of climatic suitability based on the values of MTSS [78]: unsuitability (< MTSS), low suitability (MTSS-0.4), moderate suitability (0.4–0.6), and high suitability (> 0.6) based on continuous suitability from the outputs under the background of current climate and future climatic scenarios. To examine the distribution trends for both pathogen in Eurasia, the size changes in SRs were calculated as follows [79]:

$$\mathrm{Range}\;\mathrm{size}\;\mathrm{change}=\frac{\mathrm{future}\;\mathrm{suitable}\;\mathrm{area}-\mathrm{current}\;\mathrm{suitable}\;\mathrm{area}}{\mathrm{current}\;\mathrm{suitable}\;\mathrm{area}}\times100\%$$

To compare the differences and changes in suitable habitats of *Bd* and *Bsal* between Asia and Europe, we divided the maps of habitat suitability and binary suitable habitats of Eurasia into the two regions (Europe and Asia). Binary suitable habitats in each region were used to evaluate changes in centroid positions and distributions, and these processes were completed using SDMtoolbox [80]. The area changes in reclassified habitat suitability for each region were calculated using spatial analysis tools in ArcMap v10.4 (ESRI).

To better understand the changes in the ecological niches of *Bd* and *Bsal* pathogens over time, we measured the extent of overlap between their niches and ranges. For this, we estimated their niche breadth by utilizing the inverse concentration metric of mean 'B' in ENMTools, which was based on the suitability space (cells) generated from Maxent models for both pathogens [61, 81]. Levin’s metric range was the same as *D* metric and *I* index, which ranged from 0 to 1, where 0 represents minimum niche breadth and 1 represents maximum niche breadth [82].

While MaxEnt is generally a solid approach, some authors criticize the single-algorithm strategy [83, 84]. Therefore in order to verify the robustness of our predictions we also performed an ensemble of multiple techniques in “Biomod2” package [85].We used eight algorithms, Generalized Linear Models (GLM), Generalized Boosting Models (GBM), Classification Tree Analysis (CTA), Surface Range Envelop (SRE), Flexible Discriminant Analysis (FDA), Multivariate Adaptive Regression Splines (MARS), Random Forest (RF) and Maximum Entropy (MAXENT.phillips), to evaluate their current distributions. In the modelling process, pathogen records were divided into the training data (75%) and test data (25%) and repeated 10 times; three different sets of pseudo-absences were created by randomly sampling from climatic space available within Eurasia. Out of the established 240 different models for each pathogen, 158 models of *Bd* had TSS scores > 0.6 (KAPPA_average_ = 0.66, TSS_average_ = 0.80, ROC_average_ = 0.91), 181 models of *Bsal* had TSS scores > 0.6 (KAPPA_average_ = 0.83, TSS_average_ = 0.85, ROC_average_ = 0.93). The final ensemble model was constructed to simulate and predict the suitable climatic areas at present based on the metric threshold (TSS > 0.6) [84].

## Supplementary Information

**Additional file 1.**

**Additional file 2.**

## Data Availability

The datasets supporting the conclusions of this article are included within the article and its additional files. All codes used in this study are publicly available in specific R packages as indicated in our paper.

## Abbreviations

*Bd*: *Batrachochytrium dendrobatidis*
*Bsal*: *Batrachochytrium salamandrivoransin*
RCNs: Realized Climatic Niches
SRs: Suitable ranges
CMIP6: Coupled Model Intercomparison Project Phase 6
Bio2: Mean diurnal range
Bio8: Mean temperature in wettest quarter
Bio9: Mean temperature in driest quarter
Bio14: Precipitation of driest month
Bio15: Precipitation seasonality
Bio18: Precipitation of warmest quarter
