# Supplementary material for: The two chytrid pathogens of amphibians in Eurasia—climatic niches and future expansion
Source: BMC Ecol Evol. 2023 Jun 27;23:26. doi: 10.1186/s12862-023-02132-y (PMC10294359; doi:10.1186/s12862-023-02132-y)
Supplement: Supplementary file 2 — Additional file 2. [file 12862_2023_2132_MOESM2_ESM.pdf]

Additional figures

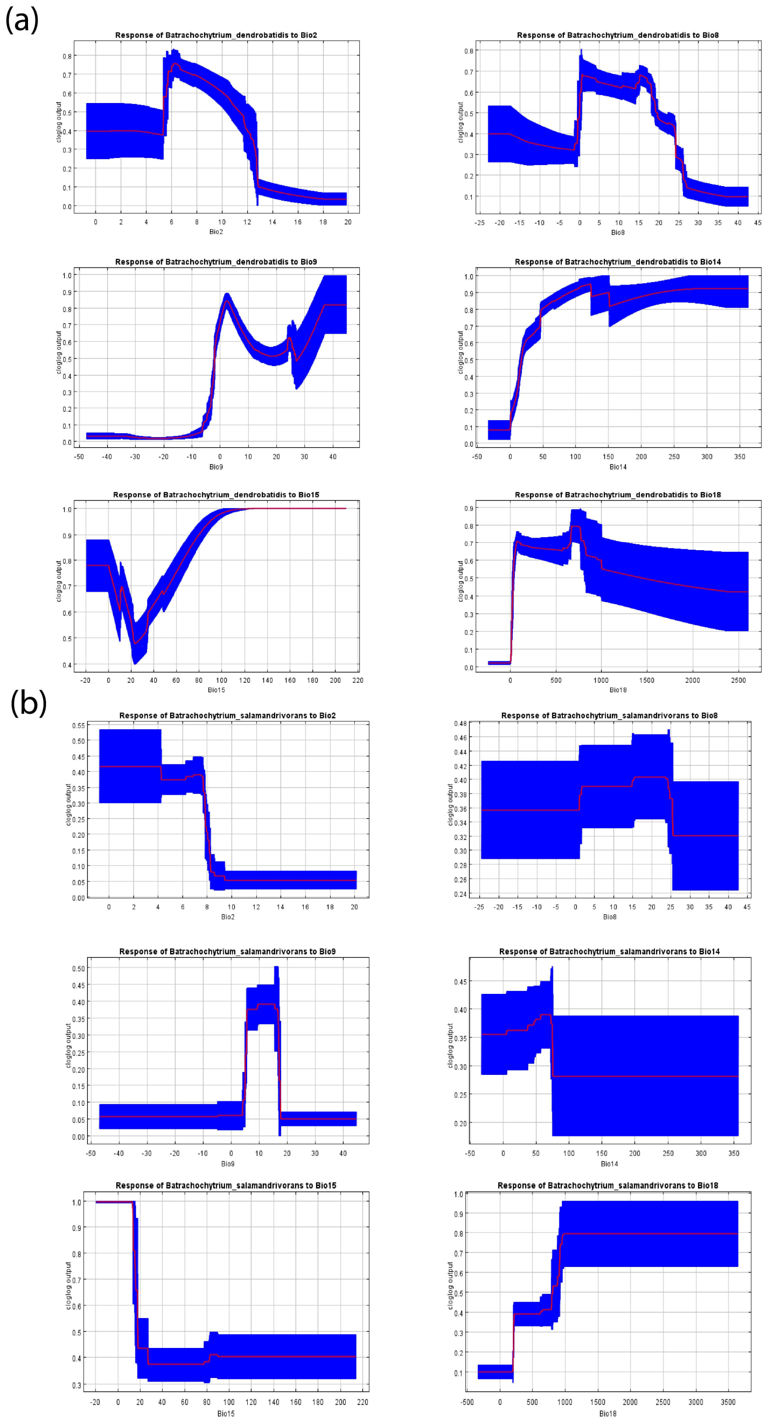

Fig. S1 Average response curves of *Bd* (a) and *Bsal* (b) to the bioclimatic variables used in the Maxent modellings.

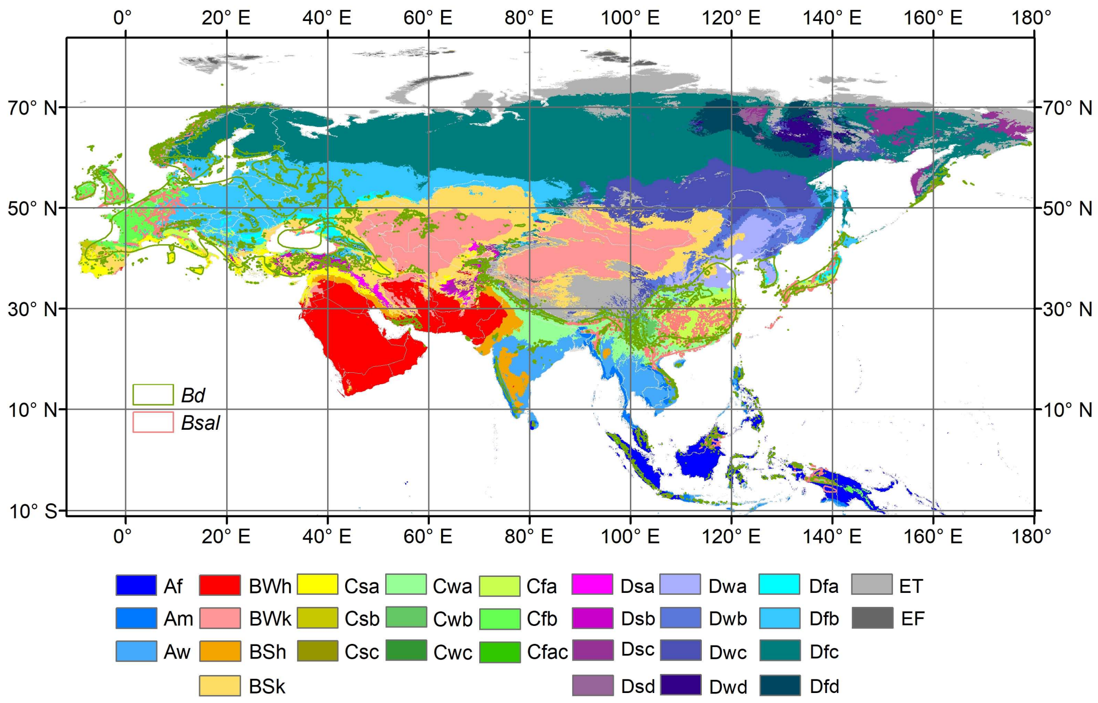

Af	Tropical, rainforest	Am	Tropical, monsoon	Aw	Tropical, savannah
BWh	Arid, desert, hot	BWk	Arid, desert, cold	BSh	Arid, steppe, hot
BSk	Arid, steppe, cold	Csa	Temperate, dry summer, hot summer		
Csb	Temperate, dry summer, warm summer	Csc	Temperate, dry summer, cold summer		
Cwa	Temperate, dry summer, hot summer	Cwb	Temperate, dry summer, warm summer		
Cwc	Temperate, dry winter, cold summer	Cfa	Temperate, no dry season, hot summer		
Cfb	Temperate, no dry season, warm summer	Cfc	Temperate, no dry season, cold summer		
Dsa	Cold, dry summer, hot summer	Dsb	Cold, dry summer, warm summer		
Dsc	Cold, dry summer, cold summer	Dsd	Cold, dry summer, very cold winter		
Dwa	Cold, dry winter, hot summer	Dwb	Cold, dry winter, warm summer		
Dwc	Cold, dry winter, cold summer	Dwd	Cold, dry winter, very cold winter		
Dfa	Cold, no dry season, hot summer	Dfb	Cold, no dry season, warm summer		
Dfc	Cold, no dry season, cold summer	Dfd	Cold, no dry season, very cold winter		
ET	Polar, tundra	EF	Polar, frost		

Fig. S2 Predicted distributions of *Bd* and *Bsal* under the background of present climate classification map. Modified climate classification map based on the new and improved Köppen-Geiger classifications from [1], the different colors representing classification that was adopted from [2].

(a)

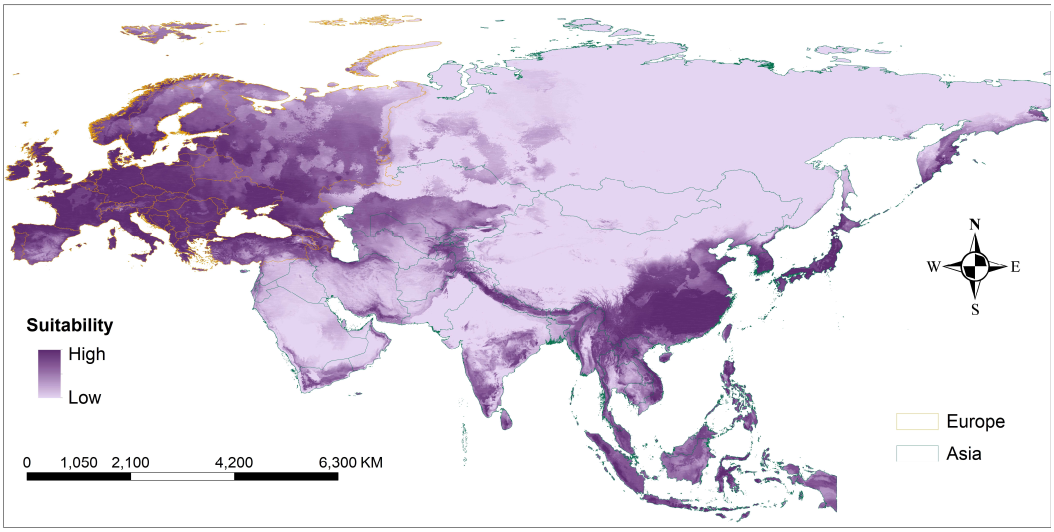

(b)

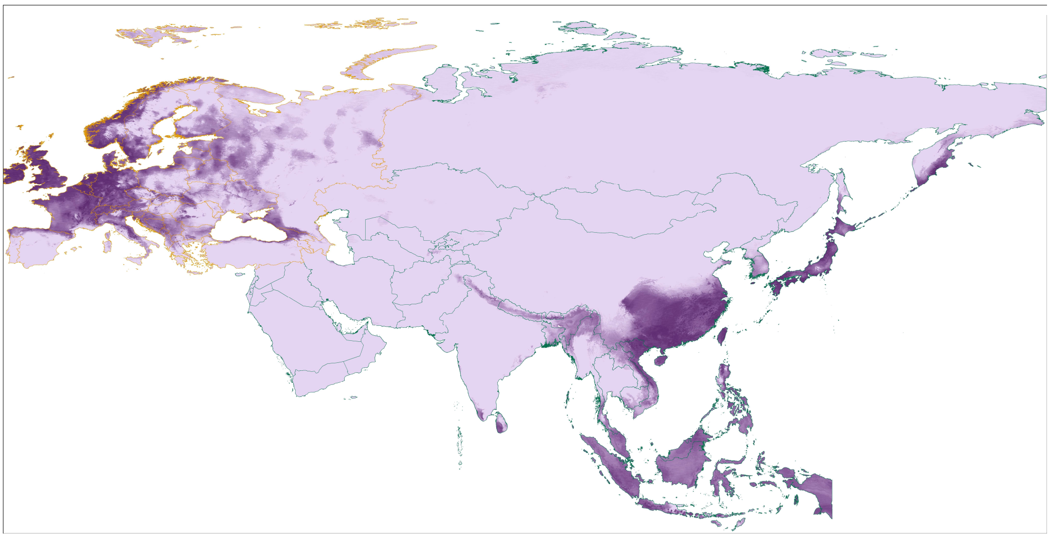

Fig. S3 Currently climatic suitability of *Bd* (a) and *Bsal* (b) generated by ensembles models.

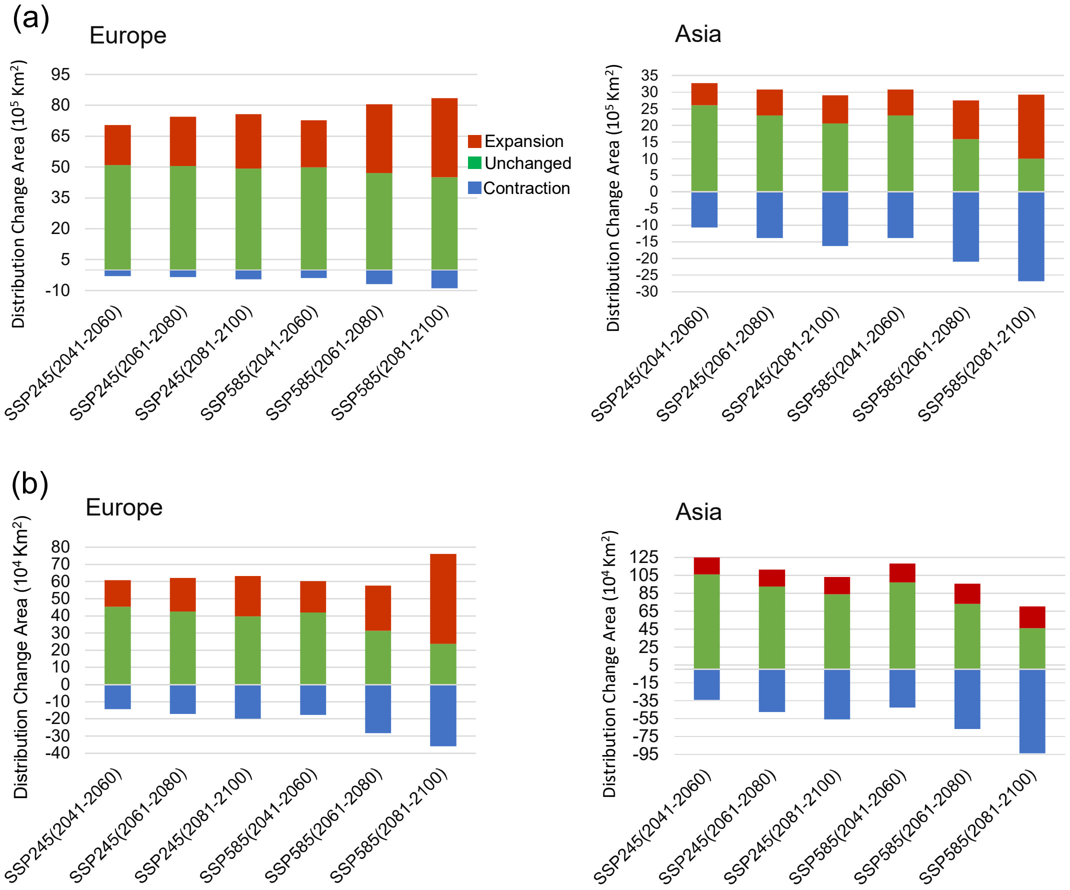

Fig. S4 Distribution changes in area of *Bd* and *Bsal*. The range size change of *Bd* (a) and *Bsal* (b) in Europe and Asia were from current to future SSP245 and SSP585 climatic scenarios during three time slices (2041-2060, 2061-2080, 2081-2100).

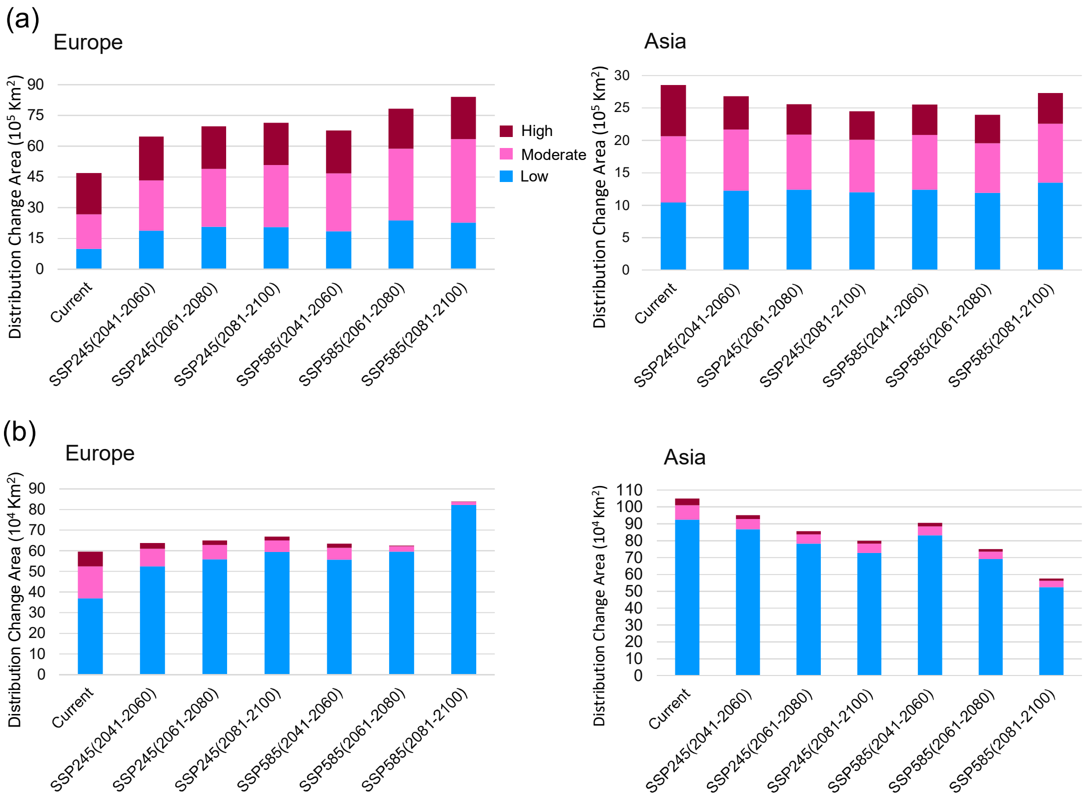

Fig. S5 Changes in the areas of climatic suitability categories for *Bd* and *Bsal*.

Climate suitability categories include: low, moderate, and high suitability. The change of distribution areas of different suitability categories for *Bd* (a) and *Bsal* (b). Changes in both Europe and Asia under SSP245 and SSP585 scenarios across three future periods (2041-2060, 2061-2080, 2081-2100) are depicted.

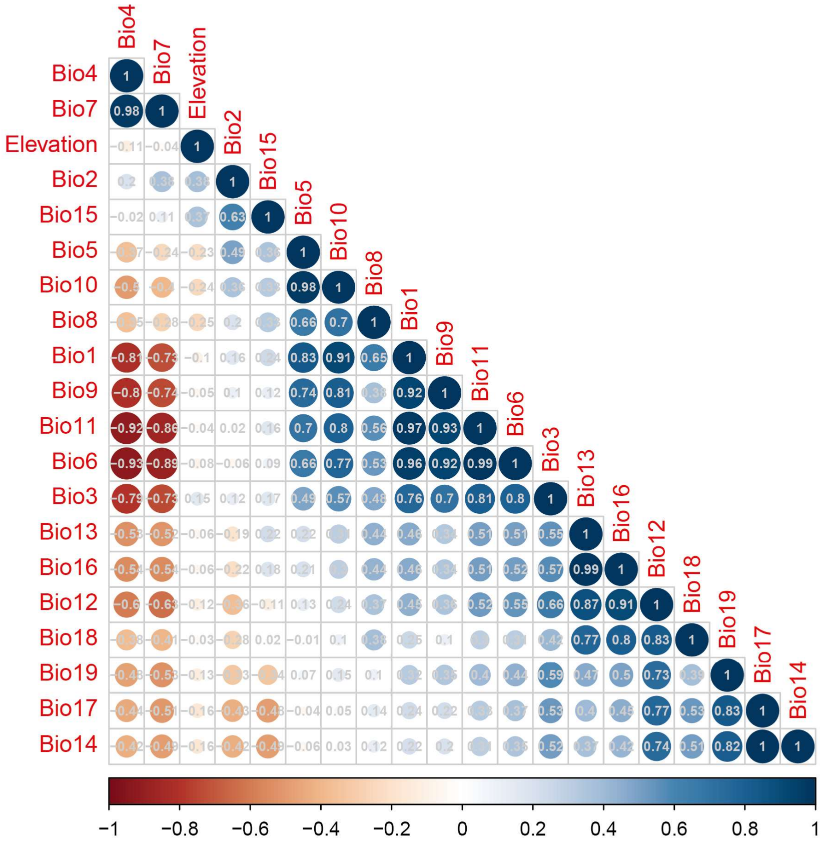

Fig. S6 Pearson pair-wise correlation among environmental variables.

References

1. Beck HE, Zimmermann NE, McVicar TR, Vergopolan N, Berg A, Wood EF. Figshare. 2018.
2. Peel MC, Finlayson BL, McMahon TA. Updated world map of the Köppen-Geiger climate classification. Hydrol Earth Syst Sci. 2007;11(5):1633-44.
